# Adaptation to an Intracellular Lifestyle by a Nitrogen-Fixing, Heterocyst-Forming Cyanobacterial Endosymbiont of a Diatom

**DOI:** 10.3389/fmicb.2022.799362

**Published:** 2022-03-17

**Authors:** Enrique Flores, Dwight K. Romanovicz, Mercedes Nieves-Morión, Rachel A. Foster, Tracy A. Villareal

**Affiliations:** ^1^Instituto de Bioquímica Vegetal y Fotosíntesis, CSIC, Universidad de Sevilla, Seville, Spain; ^2^Department of Psychology, The University of Texas at Austin, Austin, TX, United States; ^3^Department of Ecology, Environment and Plant Sciences, Stockholm University, Stockholm, Sweden; ^4^Department of Marine Science and Marine Science Institute, The University of Texas at Austin, Port Aransas, TX, United States

**Keywords:** cyanobacteria, diatom, *Hemiaulus hauckii*, heterocyst, membrane vesicles, *Richelia intracellularis*, symbiosis

## Abstract

The symbiosis between the diatom *Hemiaulus hauckii* and the heterocyst-forming cyanobacterium *Richelia intracellularis* makes an important contribution to new production in the world’s oceans, but its study is limited by short-term survival in the laboratory. In this symbiosis, *R. intracellularis* fixes atmospheric dinitrogen in the heterocyst and provides *H. hauckii* with fixed nitrogen. Here, we conducted an electron microscopy study of *H. hauckii* and found that the filaments of the *R. intracellularis* symbiont, typically composed of one terminal heterocyst and three or four vegetative cells, are located in the diatom’s cytoplasm not enclosed by a host membrane. A second prokaryotic cell was also detected in the cytoplasm of *H. hauckii*, but observations were infrequent. The heterocysts of *R. intracellularis* differ from those of free-living heterocyst-forming cyanobacteria in that the specific components of the heterocyst envelope seem to be located in the periplasmic space instead of outside the outer membrane. This specialized arrangement of the heterocyst envelope and a possible association of the cyanobacterium with oxygen-respiring mitochondria may be important for protection of the nitrogen-fixing enzyme, nitrogenase, from photosynthetically produced oxygen. The cell envelope of the vegetative cells of *R. intracellularis* contained numerous membrane vesicles that resemble the outer-inner membrane vesicles of Gram-negative bacteria. These vesicles can export cytoplasmic material from the bacterial cell and, therefore, may represent a vehicle for transfer of fixed nitrogen from *R. intracellularis* to the diatom’s cytoplasm. The specific morphological features of *R. intracellularis* described here, together with its known streamlined genome, likely represent specific adaptations of this cyanobacterium to an intracellular lifestyle.

## Introduction

Diatoms (Bacillariophyceae) are a morphologically and ecologically diverse group of algae that have a cell wall, known as a “frustule,” that incorporates silica as a main component (Round et al., 2007). The frustule consists of two halves (valves), the smaller hypotheca and the larger epitheca, that fit one into the other and can show radial or bilateral symmetry (Round et al., 2007). Although diatoms are frequently found as single cells, some can form chains of cells as in the case of *Hemiaulus hauckii* (Pyle et al., 2020). As in other eukaryotes, the ultrastructure of a diatom cell contains a nucleus, Golgi complex and abundant mitochondria, but also chloroplasts (Bedoshvili and Likhoshway, 2012). Diatoms are distributed worldwide, and in oceanic low-nutrient areas diatom-diazotrophic associations proliferate widely (Mague et al., 1974; Venrick, 1974; Villareal et al., 2012; Anderson et al., 2018; Pierella Karlusich et al., 2021), making an important contribution to CO_2_ fixation (primary productivity) supported by N_2_ fixation (diazotrophy) (Subramaniam et al., 2008; Karl et al., 2012). Some of these associations involve a filamentous, heterocyst-forming cyanobacterium, *Richelia intracellularis*, as the N_2_-fixing partner (Taylor, 1982; Villareal, 1992).

Cyanobacteria carry out oxygenic photosynthesis, and because the N_2_-fixing enzyme, nitrogenase, is sensitive to oxygen, these organisms usually separate nitrogen fixation from photosynthesis in either time or space (Flores et al., 2015). In heterocyst-forming cyanobacteria, N_2_ fixation under oxic conditions is confined to heterocysts, which are morphologically- and metabolically-differentiated cells suited for N_2_ fixation (Kumar et al., 2010). Heterocysts provide the vegetative cells of the filament with fixed nitrogen, and the vegetative cells provide the heterocysts with reduced carbon that is needed to fuel N_2_ fixation (Herrero et al., 2016). *Richelia* sp. belongs to the order Nostocales and, similar to young trichomes of *Calothrix* sp. and *Scytonema* sp. (Rippka et al., 1979), forms short filaments with terminal heterocysts (Villareal, 1991; Foster et al., 2011; Hilton et al., 2013). *Richelia intracellularis* can be found associated with diatoms such as *Hemiaulus hauckii*, *H. membranaceus* and *Rhizosolenia clevei*. In the latter, the cyanobacterium is found in a “periplasmic” location, i.e., situated between the diatom’s cytoplasmic membrane and frustule (Villareal, 1990; Janson et al., 1995), whereas in *H. hauckii* the cyanobacterium appears to be located in the cytoplasm (Caputo et al., 2019). The draft genomes of *Richelia* symbionts of *H. hauckii* and *R. clevei* differ, where the internal *Richelia* genome is far reduced containing incomplete sequences and lacking, for example, several N assimilatory pathways (Hilton et al., 2013; Nieves-Morión et al., 2020). Intracellular bacteria in eukaryotic cells can be found surrounded by a membrane of the host (as in a membrane-bound vacuole) or residing directly in the cytoplasm (Salje, 2021), but how integrated the *R. intracellularis* is to the cytoplasm of *H. hauckii* is unknown.

The cyanobacteria have a Gram stain-negative type of cell envelope in which a cytoplasmic membrane, a peptidoglycan layer and an outer membrane can be discerned (Hahn and Schleiff, 2014). The heterocysts of free-living heterocyst-forming cyanobacteria, including the model strain *Anabaena* sp. PCC 7120 (hereafter *Anabaena*), bear outside of the outer membrane an extra envelope that is composed of an inner laminated glycolipid layer (Hgl) and an outer homogeneous polysaccharide layer (Hep) (Lang and Fay, 1971; Nierzwicki-Bauer et al., 1984; Adams and Duggan, 1999; Flores et al., 2006). The Hgl layer appears to restrict oxygen diffusion into the heterocyst whereas the Hep layer seems to protect the Hgl layer from dispersal into the medium (Xu et al., 2008; Nicolaisen et al., 2009). Interestingly, *Hemiaulus*-associated *Richelia* produce distinct glycolipids that contain ribose instead of the hexose and longer aglycone moieties than those found in free-living heterocyst-forming cyanobacteria (Schouten et al., 2013; Bale et al., 2015). In the heterocysts of *Anabaena* sp., the intracellular membranes characteristic of cyanobacteria (the thylakoids that carry out photosynthesis) are concentrated close to the heterocyst poles, where they form a “honeycomb”-like structure (Lang and Fay, 1971). Terminal respiratory oxidases, which can consume oxygen that enters the heterocyst through the heterocyst-vegetative cell junctions, appear to be located at these honeycomb membranes (Murry et al., 1981; Valladares et al., 2007; Torrado et al., 2019). Heterocysts can be found intercalary or terminally in the *Anabaena* filaments. At the heterocyst pole next to a vegetative cell, the heterocyst presents a narrowing that forms the so called “heterocyst neck” (Flores and Herrero, 2010). Cyanophycin, a nitrogen reservoir composed of aspartate and arginine, accumulates within the heterocyst neck and next to it (Sherman et al., 2000).

In this work, we present an electron microscopic study of *H. hauckii* with an emphasis on its endosymbiont, *R. intracellularis*. We show that *R. intracellularis* resides directly in the cytoplasm of the diatom. Additionally, a second prokaryotic organism, which was less frequently observed, appeared also integrated in the *H. hauckii* cytoplasm. The cell envelope of *R. intracellularis* contains numerous membrane vesicles and shows a close association to the diatom host’s mitochondria. We also found that heterocyst morphology is different in *R. intracellularis* than in free-living heterocyst-forming cyanobacteria.

## Materials and Methods

This study was performed with symbiotic *Hemiaulus hauckii* cells grown from samples collected with net tows in the channel at Port Aransas, Texas, Gulf of Mexico in Fall 2012. Late exponential phase, dense cultures were prepared using an artificial seawater medium as described in Pyle et al. (2020). Cultures were grown at 25°C on a 12-h light:12-h dark cycle. From these cultures, individual short chains of diatom cells containing symbionts were isolated by micropipette under a stereomicroscope and pelleted or attached to a fluorocarbon substrate (ACLAR) with poly-L-Lys for subsequent processing.

For electron microscopy, cells were initially fixed overnight at room temperature, in a mixture of EM-grade aldehyde fixatives: 4% glutaraldehyde and 2% formaldehyde in 0.1 M cacodylate buffer at pH 7.4 with 35 mg/mL Instant Ocean. After rinses in buffer with decreasing salinity, the cells were fixed in reduced osmium (a mixture of 2% osmium tetroxide and 2% potassium ferrocyanide in 0.1 M cacodylate buffer, pH 7.4). Before additional processing, the cell preparations were dehydrated in an ethanol series, in which 2% uranyl acetate was incorporated in the 70% ethanol step. From this point there were two paths: (i) cells for scanning electron microscopy (SEM) were dehydrated with critical point drying (CPD) and then coated with 15 nm of platinum-palladium and imaged with a Zeiss Supra 40VP SEM; (ii) cells for transmission electron microscopy (TEM) were embedded in Epon Hard 812 (Electron Microscopy Sciences)^1^, from which 70-nm sections were picked up on Formvar-coated grids and imaged with a Tecnai T12 TEM, operated at 80 kV.

Measurements of cell characters were made in ImageJ (Schneider et al., 2012). Briefly, select images were imported into ImageJ, the embedded scale bar from the micrograph was used to calibrate the scale using the line tool, and three consecutive linear measurements were made for the following cell characters: heterocyst and vegetative cell dimensions (circular cross-section and cell length), frustule and spine dimensions (diameter and length), and distance between cells for a chain of *H. hauckii*. The average and standard deviation of the three measurements is reported. Additionally, the numbers of chloroplasts per *H. hauckii* were also enumerated when cross sectioning allowed full visualization.

The search for specific genes in the genome sequence of *R. intracellularis* strain HH01 (available at https://img.jgi.doe.gov/cgi-bin/m/main.cgi) was performed by BLASTp analysis (Altschul et al., 1997) using proteins from *Anabaena* sp. strain PCC 7120 as queries.

## Results

### Chains of *Hemiaulus hauckii* Cells

**FIGURE 1:**
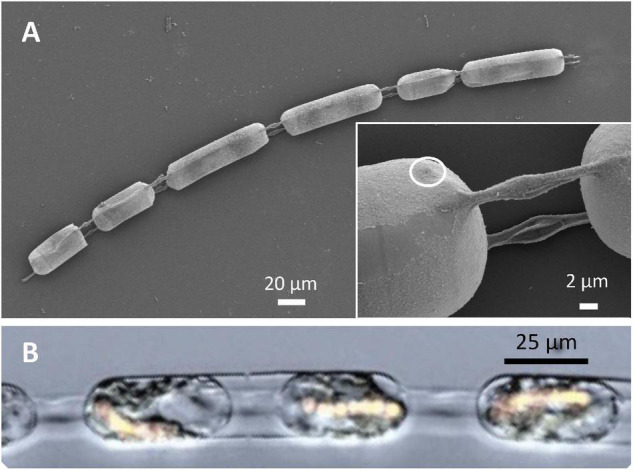
Chains of *Hemiaulus hauckii* cells. **(A)** Scanning electron micrograph of a chain of cells that are joined by thin parallel extensions (elevations) of the frustule. Inset, magnified view of elevations; the circle highlights the possible external opening of the labiate process. **(B)** Overlay of light and epifluorescence micrographs (excitation filter at 490 nm; emission, 565 nm) of part of a chain of *H. hauckii* cells (water mount) illustrating that the longer frustules in panel A can contain two diatom cells each including endosymbiotic *Richelia intracellularis* (filaments of cells with yellow-orange fluorescence).

A sample of *H. hauckii* visualized by scanning electron microscopy (SEM) showed chains of diatom cells (Figure 1). In these chains, the cells are joined by elevations with terminal spines (Hasle and Syvertsen, 1996) of the frustule; the length (15.6 ± 3.7 μm) and width (1.35 ± 0.3 μm) of the elevations varied nearly two-fold in combined observations (Figure 1 and Table 1). The distance between the cells varied from 5 to 12.5 μm. The cells in this sample were about 20 μm wide and 44–82 μm long (Table 1). The longest cells as viewed by scanning microscopy can indeed be frustules containing two cells, each of which contains a endosymbiotic cyanobacterium, *R. intracellularis* (Figure 1B). The frustule was observed to contain one large pore on its surface (Figure 1A, inset and Supplementary Figures 1A,B; pore width, about 200 nm) and numerous small pores in its internal face (Supplementary Figures 1C,D). Diatoms possess different types of pores with complex structures and function, including the labiate process and pores that can allow nutrient and waste diffusion through the frustules (Medlin et al., 1986; De Tommasi et al., 2017).

**TABLE 1:** Summary of cell measurements from scanning electron microscopy (SEM) and transmission electron microscopy (TEM) micrographs of *Hemiaulus hauckii-Richelia intracellularis* symbioses.

Cell character	Number of observations	Average diameter ± SD (min–max), μm	Average length ± SD (min-max), μm
*Hemiaulus hauckii* frustule	8	20.6 ± 0.98 (19.1–21.8)	62.3 ± 16.4 (44.2–82.1)
Cell–cell linking elevation	6	1.35 ± 0.3 (0.92–1.7)	15.6 ± 3.7 (12.7–22.7)
*Richelia* heterocyst	8	3.97 ± 0.74 (2.65–4.90)	3.42 ± 0.91 (2.36–4.82)
*Richelia* vegetative cells	69	2.62 ± 0.85 (0.67–4.41)	2.13 ± 0.87 (0.42–3.95)
Full *Richelia* filaments	6		
Vegetative cell 1		1.96 ± 0.26 (1.53–2.21)	2.44 ± 0.4 (1.99–3.0)
Vegetative cell 2		1.67 ± 0.27 (1.35–1.97)	2.21 ± 0.36 (1.82–2.75)
Vegetative cell 3		1.52 ± 0.50 (0.78–2.07)	2.06 ± 0.58 (1.26–3.03)
Vegetative cell 4		1.55 ± 0.59 (0.69–2.38)	1.63 ± 0.54 (0.92–2.29)

*Full Richelia filaments defined as four vegetative cells and one terminal heterocyst; cell 1–4, closer to farther to/from the heterocyst. SD, standard deviation.*

### *Hemiaulus hauckii* Cellular Structures

**FIGURE 2:**
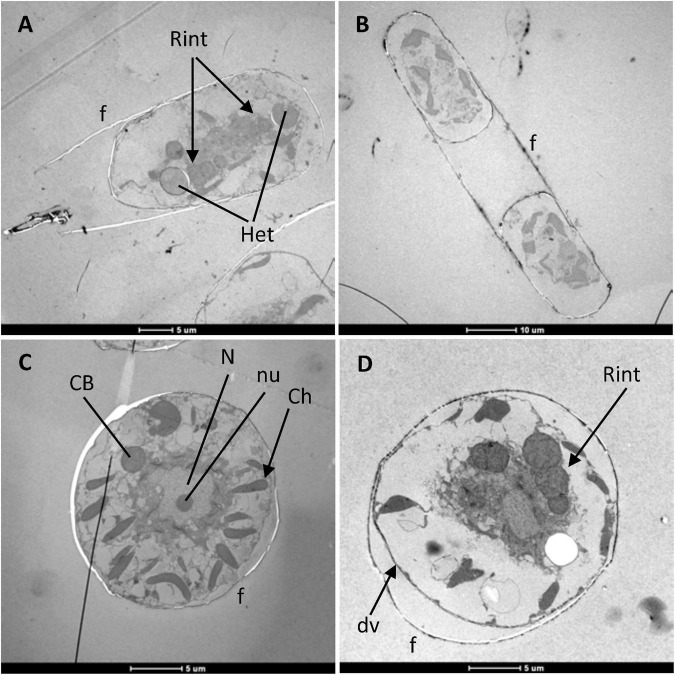
Transmission electron micrographs of *H. hauckii*. Longitudinal sections of a single cell **(A)** or of a pair of cells **(B)** and transverse sections of single cells **(C,D)** can be observed. Filaments of *R. intracellularis* (Rint) can be seen in panels **(A,D)** (Het, heterocyst), and a transverse section of a cyanobacterial cell (CB) can be seen in **(C)** (this cell is likely a vegetative cell of a *R. intracellularis* filament). Additionally, some diatom structures are evident: frustules (f) can be seen as the outermost layers in the cells of the four micrographs, a developing valve can be seen in the lower left part of the cell in panel **(D)** (dv), and the nucleus (N), including the nucleolus (nu), and several chloroplast lobes (Ch) are evident in the diatom cell in panel **(C)**.

Samples of *H. hauckii* were prepared for transmission electron microscopy (TEM). Longitudinal (Figures 2A,B) and transverse (Figures 2C,D) sections of single diatom cells or pairs of cells were frequently observed. As expected, the diatoms were surrounded by their frustule, and in some micrographs internal structures such as the nucleus (including the nucleolus), mitochondria, Golgi bodies, and numerous chloroplasts or chloroplast lobes (8 ± 2 per *H. hauckii*, n = 20; Figure 2C and Supplementary Figure 2) were evident. A developing valve can be observed in the lower-left part of the diatom cell in Figure 2D, indicating that it is a cell in the process of division. Magnification of an area including the internal valve showed that it is within a membrane vesicle (Supplementary Figures 3A,B), suggesting that it is indeed a recently synthesized valve that is still within the silicalemma or silica-deposition vesicle (Crawford, 1981; Hildebrand et al., 2018). A magnified view of an area including chloroplasts showed the presence of plastoglobules (lipid droplets) in one of them (Supplementary Figure 2B). Other than the diatom structures, cyanobacterial cells or filaments were observed in most diatom cells (e.g., Figures 2A,C,D and Supplementary Figure 2A), and we did not generally notice the presence of other bacterial endosymbionts (see, however, below).

### *Richelia intracellularis* Resides in the *Hemiaulus hauckii* Cytoplasm

**FIGURE 3:**
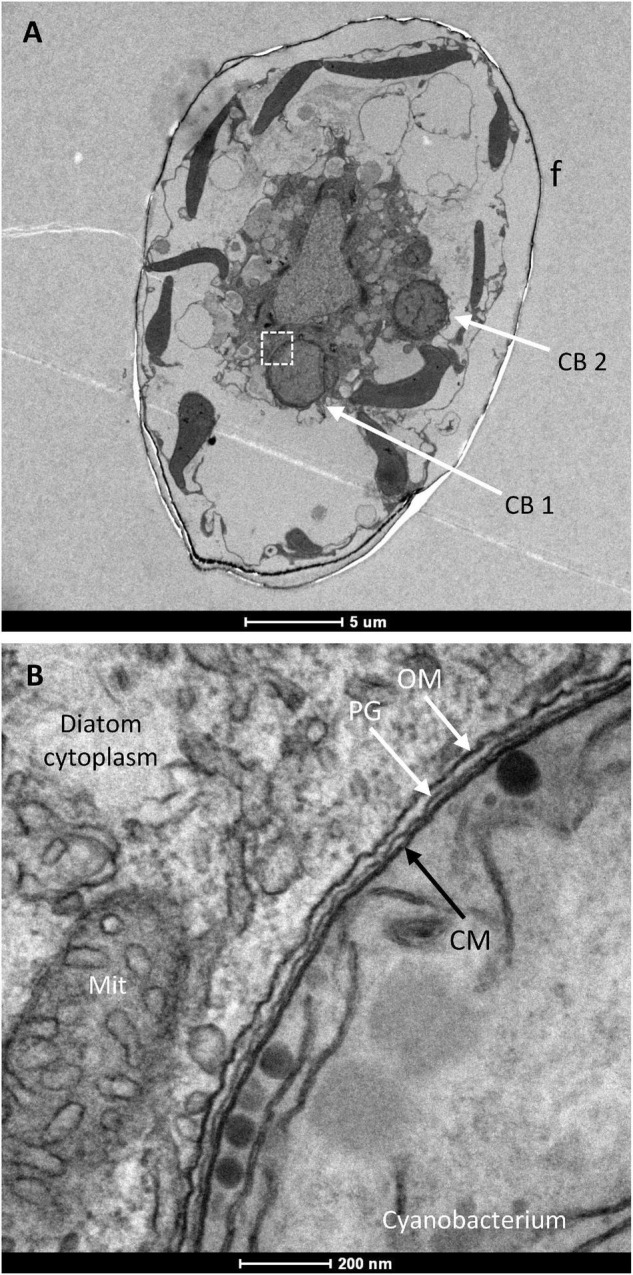
Transmission electron micrograph of a *H. hauckii* cell. **(A)** Transverse section of the diatom containing two cyanobacterial cells (CB) also mainly in transverse section; f, diatom frustule. **(B)** Magnified view of the diatom cytoplasm and part of the cyanobacterium indicated by the dotted square in panel **(A)**. Note components of the cyanobacterial cell envelope: CM, cytoplasmic membrane; PG, peptidoglycan layer; OM, outer membrane. Note also the mitochondrion (Mit) close to the cyanobacterial cell.

**FIGURE 4:**
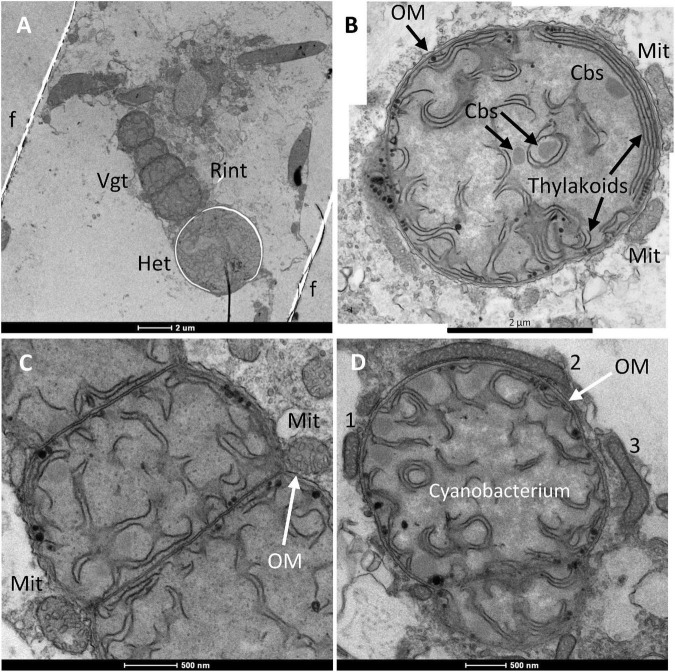
Transmission electron micrograph of mitochondria and *R. intracellularis* in the cytoplasm of *H. hauckii*. **(A)** Longitudinal section of a *R. intracellularis* filament (Rint) composed of one terminal heterocyst (Het) and four vegetative cells (Vgt). The cyanobacterial filament is located within a diatom cell; f, frustule. **(B)** Transverse section of a cyanobacterial cell with mitochondria (Mit) next to the outer membrane (OM). Note also the presence of carboxysomes (Cbs) and thylakoids (intracellular membranes) in the cyanobacterium. **(C)** Magnified view of a section of the filament from panel **(A)** containing vegetative cells and mitochondria (Mit) nearby; OM, outer membrane. **(D)** Transverse section of a *R. intracellularis* cell showing nearby mitochondria (some indicated by numbers, 1–3). Note that mitochondria 1 and 2 are tightly associated to the outer membrane of the cyanobacterium, whereas mitochondrion 3 is not bound to the cyanobacterium.

We next focused on the cyanobacteria that could be observed within the diatom cells. As shown in Figure 1B, *H. hauckii* contains filaments of the heterocyst-forming cyanobacterium *R. intracellularis*. Depending on the orientation of the specimen, transverse sections of cyanobacterial cells (Figure 3A) or longitudinal sections of cyanobacterial filaments (Figure 4A) could be observed. The cyanobacterial filament CB 1 in Figure 3A is somewhat tilted so that a transverse section shows a whole cell and a fragment of its adjacent cell. Twenty-eight *H. hauckii* cells were imaged containing one to three filaments of *R. intracellularis* per diatom cell (1.3 ± 0.6 [mean ± SD]). In total, 34 *R. intracellularis* filaments were measured for cell dimensions of the heterocyst and vegetative cells. Considering the dimensions summarized in Table 1, the heterocysts were about 2.5- to 3-fold larger in volume than an average vegetative cell. The number of vegetative cells per filament was 1.9 ± 1.2 (mean ± SD), which, considering that sectioning of tilted filaments may hide some of the cells in the TEM, is consistent with earlier reports of 3.5 cells per filament found by fluorescence microscopy (Caputo et al., 2019; Tuo et al., 2021). In the filaments containing three or four vegetative cells, tapering of the filament was evident as the size of the cells decreased along the filament, with the smallest cell being located farthest from the terminal heterocyst (Figure 4A and Table 1). Because filaments could be somewhat tilted in the sectioning plane, tapering observed by TEM should be taken with caution; however, this observation is consistent with tapering of the *R. intracellularis* filaments visualized by fluorescence microscopy (Hilton et al., 2013; Pierella Karlusich et al., 2021). Taking as reference a filament of *R. intracellularis* made of one heterocyst and four vegetative cells (see cell dimensions in Table 1) and a standard *H. hauckii* cell as viewed by TEM (see Figure 2: length ca. 25–30 μm, diameter ca. 15–20 μm; considered as a cylinder to simplify calculations), we estimate that one cyanobacterial filament occupies about 1% of the volume of the host diatom.

The cyanobacterial cell envelope, comprising the cytoplasmic membrane, peptidoglycan layer and outer membrane, was very well defined in many images (e.g., Figure 3B). Additionally, thylakoids (photosynthetic intracellular membranes) were generally observed, and carboxysomes (CO_2_-fixing structures) were clearly observed in some images (e.g., Figure 4B). Abundant glycogen granules were observed only in few images (Supplementary Figure 4), indicating that the corresponding *H. hauckii-R. intracellulais* cells were in a different physiological state than the other cells that were visualized that lacked such a large number of glycogen granules. In contrast, no evidence was found for the presence of gas vesicles, which are common in other oceanic filamentous cyanobacteria (Hynes et al., 2012).

Independently of the section, mitochondria were frequently observed close to the cyanobacterium (Figures 3B, 4B–D), more frequently associated with the vegetative cells than with heterocysts and sometimes accommodated nearby the cyanobacterial filament in the zone of the intercellular septa (Figure 4C). In some instances, the mitochondria were tightly associated to the cyanobacterial outer membrane (Figure 4D and see Supplementary Figure 6 below). In spite of an excellent definition of membranes, no membrane was observed in any case surrounding a cyanobacterial cell outside of the outer membrane or between the outer membrane and a nearby mitochondrion. These observations strongly support the idea that *R. intracellularis* resides directly in the cytoplasm of *H. hauckii*.

### *Richelia intracellularis* Envelope Vesicles

**FIGURE 5:**
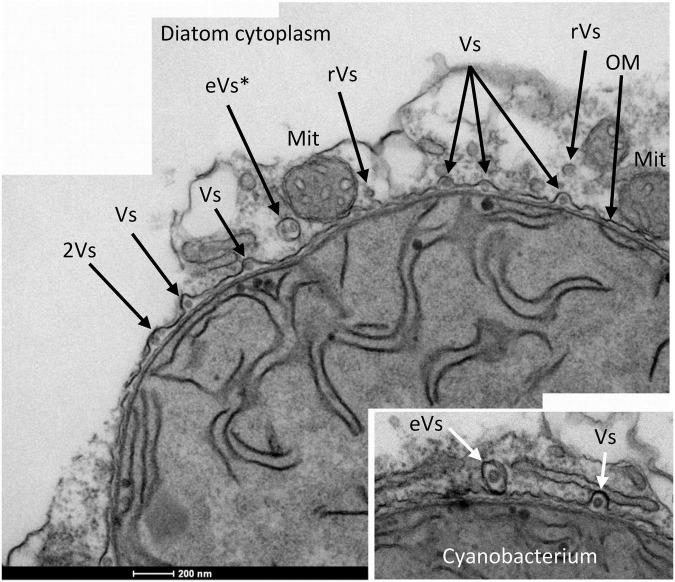
Transmission electron micrographs of sections of a vegetative cell of *R. intracellularis* endosymbiotic in *H. hauckii* showing numerous cell envelope-related vesicles. Periplasmic vesicles mainly located in outer membrane evaginations (Vs) and possible recently released vesicles (rVs) can be observed. Two closely located vesicles (2Vs) could be externalized together giving rise to an extruded vesicle such as that shown with two internal vesicles (eVs*). Two mitochondria nearby the cyanobacterial outer membrane (OM) are also indicated (Mit). In the lower-right micrograph, a possible extruded vesicle containing one internal vesicle is observed (eVs).

A distinct feature observed in the vegetative cells of *R. intracellularis* was the presence of vesicles in their periplasmic space (Figure 5 and see also Supplementary Figure 5). These structures are about 40–60 nm in diameter, and their vesicular nature is evident as they appear to be composed of a closed bilayer. Their localization in the periplasm suggests that these vesicles originate from the cytoplasmic membrane. These vesicles are mainly found outside of the peptidoglycan layer and within partial evaginations of the outer membrane. Outside of the outer membrane, some double vesicles about 100 nm × 150 nm (eVs) or 115 nm in diameter (eVs*) that consist of one or two vesicles surrounded by a membrane were observed (Figure 5). These double vesicles may represent vesicles that have been extruded from the cyanobacterium surrounded by outer membrane. Finally, some small vesicles observed in the diatom’s cytoplasm close to the cyanobacterium (rVs) might correspond to released periplasmic vesicles (Figure 5 and Supplementary Figure 5). If this were the case, released vesicles with one membrane (rVs) could represent extruded vesicles that shed the outer membrane layer.

### Distinct Heterocyst Morphology

**FIGURE 6:**
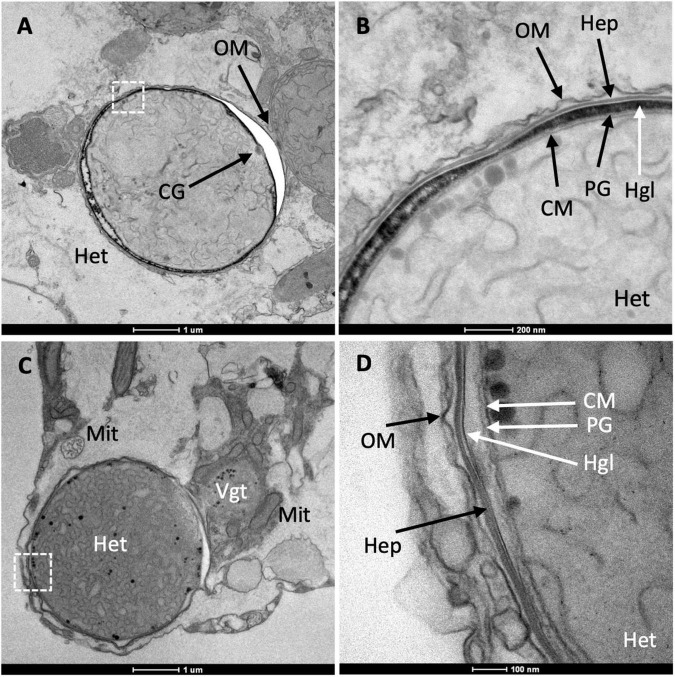
Transmission electron micrographs of *R. intracellularis* heterocysts and details of the heterocyst envelope. **(A)** Magnified view of the left-hand heterocyst (Het) in Figure 2A. Note the outer membrane (OM), which is continuous with that of the adjacent vegetative cell, and the presence of a possible cyanophycin granule (CG); the relative accumulation of membranes close to the CG resembles the heterocyst honeycomb structure. **(B)** Magnified view of part of the envelope from the heterocyst shown in panel **(A)** (dotted square). Note the presence of cytoplasmic membrane (CM) and outer membrane (OM). Three additional layers tentatively identified as peptidoglycan layer (PG), glycolipid layer (Hgl) and polysaccharide layer (Hep) are shown. **(C)** Part of a diatom cell in which a heterocyst (Het) and part of an adjacent vegetative cell (Vgt) can be observed. Note the presence of nearby mitochondria (Mit). **(D)** Magnified view of part of the envelope from the heterocyst shown in panel **(C)** (dotted square). Heterocyst envelope layers tentatively identified as cytoplasmic membrane (CM), peptidoglycan layer (PG), glycolipid layer (Hgl), polysaccharide layer (Hep) and outer membrane (OM) are indicated. Note that the putative glycolipid layer is thinner in this image than in that shown panels **(A,B)**.

**FIGURE 7:**
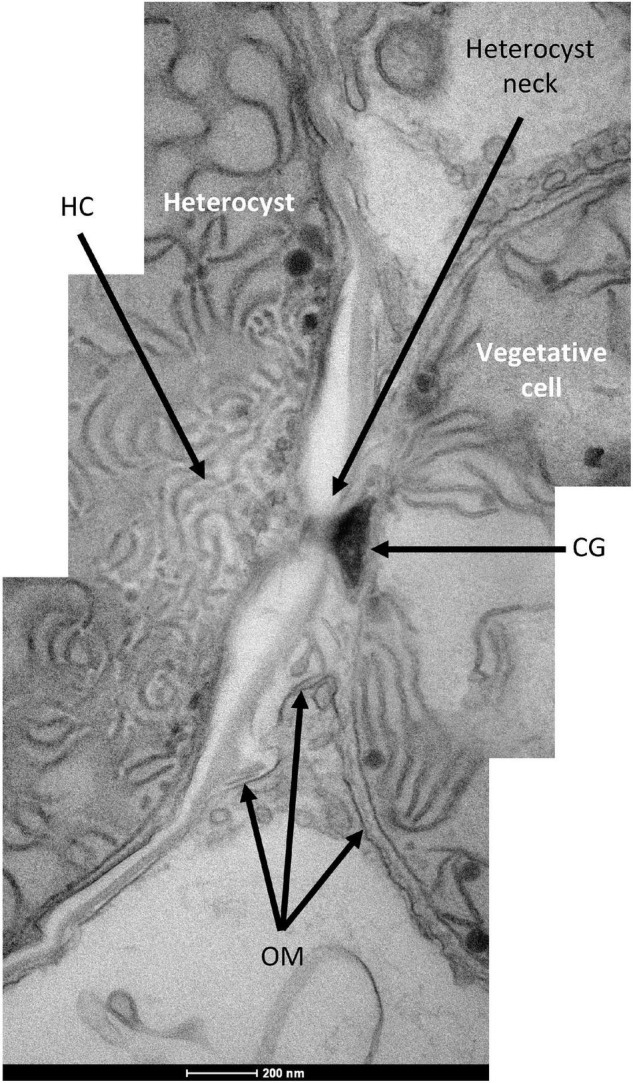
Transmission electron micrograph of the vegetative cell-heterocyst junction in a *R. intracellularis* filament endosymbiotic in *H. hauckii.* Note the honeycomb membrane structure (HC), the heterocyst neck, the continuous outer membrane (OM) and the cyanophycin granule (CG).

In the *Richelia* filaments shown in Figures 2A, 4A, the heterocysts present a cell envelope that is thinner than that usually observed in free-living heterocyst-forming cyanobacteria such as *Anabaena*, about 100 nm wide in *R. intracellularis* vs. 250–600 nm wide in *Anabaena* (see, e.g., Flores et al., 2019). In contrast, the envelope of the vegetative cells is about 50 nm wide in both *R. intracellularis* (Figures 3B, 5) and *Anabaena* (Wilk et al., 2011). The blank space around a heterocyst, as seen in Figure 4A, is a frequent artifact that, in the heterocysts of *Anabaena*, results from separation of the Hgl layer from the outer membrane during sample preparation (see, e.g., Flores and Herrero, 2010; Flores et al., 2019). Other examples of heterocysts that show a relatively thin, but well labeled envelope, are shown in Figures 6A,C. On the other hand, in contrast to *Anabaena* heterocysts in which intracellular membranes tend to concentrate at the cell poles forming the honeycomb structure next to the heterocyst neck (see, e.g., Flores and Herrero, 2010; Flores et al., 2019), the *Richelia* heterocysts frequently showed intracellular membranes throughout (Figures 4A, 6C). Nonetheless, the heterocyst in Figure 6A partially shows a honeycomb structure as well as a cyanophycin granule, typically found nearby the heterocyst neck. Likely because *R. intracellularis* occupies a small volume, a clear honeycomb structure and heterocyst neck, including the cyanophycin granule, were observed only in one specimen (Figure 7). This observation indicates the presence of these structures in the heterocysts of *R. intracellularis*.

In *R. intracellularis*, the outer membrane is continuous between vegetative cells (Figure 4C and Supplementary Figures 6A,B) and between the heterocyst and its adjacent vegetative cell (Figures 6A, 7 and Supplementary Figure 7A), as is usual in heterocyst-forming cyanobacteria (Flores et al., 2006, 2016; Wilk et al., 2011). As observed in different samples, the *R. intracellularis* heterocyst envelope contains five layers including the cytoplasmic and outer membranes. Importantly, the continuity of the outer membrane with that of the adjacent vegetative cell shows that the outer membrane is the outer-most layer of the heterocyst envelope in *R. intracellularis*. In addition to the peptidoglycan layer, two additional layers of unknown composition can be discerned next to the cytoplasmic membrane (more clearly seen in Figure 6D than in Figure 6B and see also Supplementary Figures 7B,D). Because the staining procedure used was based on reduced osmium tetroxide (see “Materials and Methods”), which labels lipids efficiently (Tapia et al., 2012), we hypothesize that the sharply labeled layer contains lipids and, therefore, could constitute the heterocyst glycolipid layer (tentatively marked as Hgl in Figure 6 and Supplementary Figure 7). The layer next to, inside the outer membrane, could constitute the heterocyst polysaccharide layer (tentatively marked as Hep in Figure 6 and Supplementary Figure 7). Thus, in contrast to the envelope of the heterocysts of free-living heterocyst-forming cyanobacteria, the heterocysts of *R. intracellularis* lack an extra envelope outside of the outer membrane. Instead, the components of the heterocyst-specific envelope seem to be located in the periplasmic space, although this hypothesis will need corroboration.

### Possible Third Partner in the *Hemiaulus hauckii* Symbiosis

**FIGURE 8:**
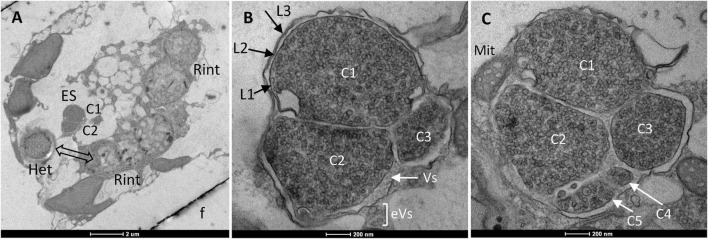
Transmission electron micrographs of a third partner in *H. hauckii*. **(A)** Diatom cell showing *R. intracellularis* filaments (Rint), a heterocyst (Het) and an unidentified additional cytoplasmic partner (tentatively denoted endosymbiont, ES) with two cells (C1, C2); f, frustule. As observed in other sectioning planes, the heterocyst (Het) belongs to the filament indicated by a double arrow. **(B,C)** Magnified view of the second endosymbiont as observed in different sectioning planes. In panel **(B)**, the presence of three cells (C1–C3) and three possible envelope layers (L1–L3) are indicated. In panel **(C)**, the presence of five differently-sized cells (C1–C5) and of a mitochondrion adjacent to layer L3 are indicated. Note the presence of vesicles inside the cells, as well as in the periplasmic space (Vs in panel **B**) and outside of L3 (eVs in panel **B**).

In one *H. hauckii* cell, a second possible prokaryotic organism was observed located in the cytoplasm (Figure 8A). It consisted of a group of cells with different sizes, and the number of cells that could be seen depended on the sectioning plane. Thus, two cells are seen in panel A, three in panel B, and five in panel C (Figure 8; see full series of images of this specimen in Supplementary Figure 8). The whole group of cells is remarkably small, about 1.8-μm wide. Assuming a spherical form, this group of cells is estimated to represent only about 0.03% of the host cell volume. The ultrastructure of this organism (Figure 8) resembles that of cyanobacteria of the genus *Chroococcidiopsis*, which reproduce by both binary and multiple fission producing cells of different sizes (Waterbury and Stanier, 1978; Billi et al., 2001). Three envelope layers could be discerned (Figure 8B), suggesting a Gram-negative type of cell envelope. This is consistent with the possibility that this is a cyanobacterium in which an outer membrane (layer L3; Figure 8B) is shared by all the cells in the group. Unlike cyanobacteria, however, this organism contains, instead of typical thylakoids, cytoplasmic vesicles that are about 50 nm in diameter and resemble the chromatophores of photosynthetic bacteria such as *Rhodobacter sphaeroides* (Noble et al., 2018). As in the case on *R. intracellularis*, a mitochondrion is observed tightly associated to the outer membrane of this second cytoplasmic organism (Figure 8C). Additionally, some vesicles are observed in the periplasmic space and outside of the cell group (Figure 8B), resembling the vesicles described earlier for *R. intracellularis*.

## Discussion

**FIGURE 9:**
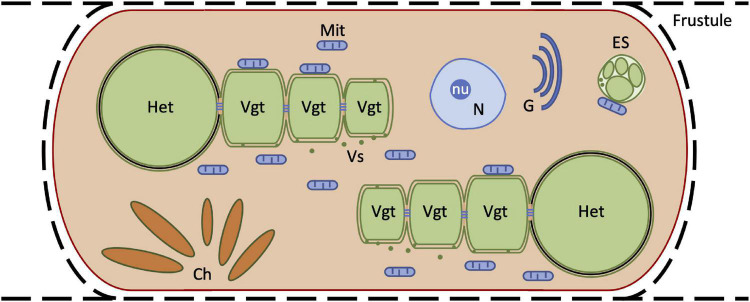
Scheme of a symbiotic *Hemiaulus hauckii* cell. Two endosymbiotic filaments of *Richelia intracellularis* composed of one terminal heterocyst (Het) and three vegetative cells (Vgt) are represented, with emphasis in the distinction between cytoplasmic (inner) and outer membranes. The black line between these membranes in the heterocyst represents heterocyst-specific components of the cell wall that in *Richelia intracellularis* are periplasmic. Thin horizontal lines between cells represent the septal junctions that mediate intercellular molecular exchange in the cyanobacterial filament. Small green dots represent membrane vesicles (Vs), which may mediate transfer of cytoplasmic material from the cyanobacterium to the diatom. Internal structures of the cyanobacterial cells are not depicted. Numerous mitochondria (Mit) are present in the cytoplasm of the diatom, and some of them are tightly associated to cyanobacterial cells. A possible second endosymbiont (ES) with an associated mitochondrion is also depicted. The frustule, a nucleus (N) including a nucleolus (nu), Golgi bodies (G) and numerous chloroplast lobes (Ch) are also shown for the diatom. Depicted structures are not to scale.

In this work, we have shown that in the *Hemiaulus hauckii-Richelia intracellularis* symbiosis, the endosymbiont retains the Gram-negative type of cell envelope characteristic of cyanobacteria and resides directly in the cytoplasm of the diatom (Figure 9). *Hemiaulus hauckii* can also host a second prokaryotic organism in its cytoplasm that shows some resemblance to cyanobacteria of the genus *Chroococcidiopsis*. Because of its small estimated size and the nature of TEM, these other cells could have been largely undetected in our samples, which unfortunately makes estimating the frequency of its presence difficult. Thus, we do not know whether these observations represent an occasional associate of *H. hauckii* (e.g., a pathogen) or an established third partner in the symbiosis. Interestingly, visualization by epifluorescence microscopy has shown the presence of a possible second non-filamentous cyanobacterial endosymbiont in *H. hauckii* collected from the same location as the cells presented here (see small fluorescence spot apart from the fluorescent *Richelia* filaments in Figure 1B of Hilton et al., 2013) as well as from a different location (Supplementary Figure 9). To the best of our knowledge, these observations represent the first report of a possible additional partner in these globally distributed symbioses, and thus warrant further investigation.

The location of *R. intracellularis* in the cytoplasm of *H. hauckii* should influence the mechanism(s) of molecular exchange between the two partners. The cyanobacterium fixes CO_2_ and N_2_ (Foster et al., 2021), and it has to obtain nutrients necessary for its growth from the diatom’s cytoplasm. A recent review of membrane transporters encoded in the genome of *R. intracellularis* HH01, an endosymbiont of a *H. hauckii* that was isolated from the same location as the *H. hauckii* studied here (Hilton et al., 2013), has identified a number of putative transporters for sugars, amino acids, sulfur, phosphorus, metals and vitamins that can support the growth of the cyanobacterium (Nieves-Morión et al., 2020). *Richelia intracellularis* provides *H. hauckii* with fixed nitrogen (Foster et al., 2011), and therefore mechanism(s) for molecular transfer (in particular, of fixed nitrogen) from the cyanobacterium to the diatom must also exist. However, possible membrane exporters encoded in the genome of *R. intracellularis* HH01 were not as evident or numerous as membrane importers (Nieves-Morión et al., 2020), suggesting the need for another strategy of metabolite export. Here, we have shown that *R. intracellularis* produces cell envelope vesicles, readily observed in the periplasm of vegetative cells before being presumably externalized surrounded by a fragment of the outer membrane. These vesicles resemble the outer-inner membrane vesicles of Gram-negative bacteria which can carry cytoplasmic material (Gill et al., 2019; Toyofuku et al., 2019). We hypothesize that the membrane vesicles surrounding *Richelia* can contribute to the transfer of nutrients (especially fixed nitrogen) from the cyanobacterium to its host. Interestingly, membrane vesicles have been observed in the proximity of the periplasmic symbiont of the diatom *Rhizosolenia clevei* (Janson et al., 1995) and a cyanobacterial symbiont in a lichen (Peveling, 1973). Membrane vesicles have also been suggested to be involved in export of cytoplasmic material from *Anabaena* cells to the growth medium (Oliveira et al., 2015). Outer-inner membrane vesicles can also carry macromolecules including nucleic acids (Gill et al., 2019), and membrane vesicles containing DNA have been observed in the cyanobacterial symbiont of the fern *Azolla* (Zheng et al., 2009). The size of the genome of *R. intracellularis* HH01 is about 3.24 Mbp, much smaller than the genome size of most free-living heterocyst-forming cyanobacteria, which is about 7–9 Mbp (Hilton et al., 2013). If some cyanobacterial genes have been transferred to the diatom nucleus, as has happened in the development of endosymbiont-derived organelles (Bock, 2017), the membrane vesicles could represent a vehicle for gene transfer.

N_2_ fixation is very sensitive to oxygen, and in *R. intracellularis* the N_2_ fixation machinery is likely exposed not only to ambient oxygen but also to oxygen produced photosynthetically by both its vegetative cells and host diatom. Here we have observed the presence of numerous mitochondria nearby the cyanobacteria in the diatom’s cytoplasm. Although this might reflect the presence of a high number of mitochondria in the diatom, mitochondria frequently appeared to be tightly associated to the outer membrane of the cyanobacterium, revealing a possible specific relation. It is possible that the mitochondria contribute to decreasing the level of intracellular oxygen in the vicinity of the cyanobacterium. Whether the endosymbiont could additionally interact with the host mitochondria as has been shown for some endosymbiotic algae (Song et al., 2017; Uwizeye et al., 2021) and for the tick bacterial symbiont *Midichloria mitochondrii* (Stavru et al., 2020), or alter the metabolism of the mitochondria as has been described for pathogenic bacteria that target the organelles of eukaryotic cells (Escoll et al., 2016), is unknown.

The heterocysts of *R. intracellularis* lack the extra envelope deposited outside of the outer membrane that is characteristic of the heterocysts of free-living heterocyst-forming cyanobacteria (Lang and Fay, 1971; Flores et al., 2006; Nicolaisen et al., 2009). Instead, the heterocysts of *R. intracellularis* appear to contain specific envelope components in the periplasm, between the cytoplasmic and outer membranes, making a heterocyst periplasm that is different from that of the vegetative cells. Because the genome of *R. intracellularis* HH01 contains most of the genes that are necessary in *Anabaena* for production of the Hep and Hgl layers (Supplementary Tables 1, 2), it is possible that the layers (other than the peptidoglycan layer) observed in the heterocyst periplasm constitute the *R. intracellularis* Hep and Hgl layers. However, the *hep* and *hgl* gene complement of *R. intracellularis* HH01 is not identical to that of *Anabaena* (Supplementary Tables 1, 2), and therefore some differences in Hep and Hgl composition may be expected, as has already been shown for the especial glycolipids produced by *Hemiaulus*-associated *Richelia* (Schouten et al., 2013). If the extra periplasmic layers observed in the heterocysts of *R. intracellularis* are indeed the Hep and Hgl layers of this cyanobacterium, the genetic basis for deposition of these layers in the periplasmic space is still unknown. The role of the Hep layer protecting the Hgl layer from dispersion in the diluted external medium in free-living organisms might be unsuitable for the intracellular location in which *R. intracellularis* thrives. Thus, the possible specific arrangement in the periplasmic space of the heterocyst Hep and Hgl layers may represent an adaptation to the intracellular lifestyle of *R. intracellularis*, impacting the protection of nitrogenase from oxygen.

The membrane vesicles that may be involved in molecular transfer from *R. intracellularis* to the cytoplasm of *H. hauckii* have been observed only in vegetative cells, raising the question of why are they missing from heterocysts. Intercellular molecular exchange in filamentous cyanobacteria takes place through proteinaceous septal junctions (Flores et al., 2016; Kieninger and Maldener, 2021). Although the microscopy used in this study is not optimal to visualize the septal junctions, their presence could be detected (Supplementary Figure 6C). Intercellular molecular exchange in the filament of *R. intracellularis* can make the vegetative cells a platform for the transfer of fixed nitrogen to the diatom. This indirect route of transfer of the nitrogen fixed in the heterocyst to the cytoplasm of the diatom may be needed because the specialized cell envelope of the heterocyst may impede the formation and export of membrane vesicles.

In summary, in the *H. hauckii-R. intracellularis* symbiosis the cyanobacterium is not enclosed by a host membrane and resides directly in the cytoplasm of the diatom. Lack of extra membranes around the cyanobacterium indicates that this is not an organelle resulting from serial endosymbiosis. However, after 50–100 million years since it was established (Caputo et al., 2019), the endosymbiont appears to have streamlined its genome (Hilton et al., 2013) and adapted to an intracellular lifestyle. Thus, the endosymbiont has developed a specialized biochemistry exemplified by the presence of specific glycolipids (Schouten et al., 2013) and the lack of central metabolic enzymes such as glutamine-oxoglutarate aminotransferase (Hilton et al., 2013). As shown in this work, the endosymbiont has also developed specialized structures for an intracellular location such as a characteristic heterocyst envelope and abundant membrane vesicles produced by the vegetative cells. The genomic reduction of the endosymbiont coupled with the direct cytoplasmic contact indicates a deep integration of host and endosymbiont, suggesting that the host is tightly regulating the physiology of the endosymbiont.

## Data Availability Statement

The original contributions presented in the study are included in the article/Supplementary Material, further inquiries can be directed to the corresponding author.

## Author Contributions

TV designed the research. TV and DR performed the research. EF, MN-M, and RF analyzed the data. EF wrote the manuscript. TV, DR, MN-M, and RF provided the manuscript revisions. All authors contributed to the article and approved the submitted version.

## Conflict of Interest

The authors declare that the research was conducted in the absence of any commercial or financial relationships that could be construed as a potential conflict of interest.

## Publisher’s Note

All claims expressed in this article are solely those of the authors and do not necessarily represent those of their affiliated organizations, or those of the publisher, the editors and the reviewers. Any product that may be evaluated in this article, or claim that may be made by its manufacturer, is not guaranteed or endorsed by the publisher.

## Footnotes

1
https://www.emsdiasum.com
