# Supplementary material for: Adaptation to an Intracellular Lifestyle by a Nitrogen-Fixing, Heterocyst-Forming Cyanobacterial Endosymbiont of a Diatom
Source: Front Microbiol. 2022 Mar 17;13:799362. doi: 10.3389/fmicb.2022.799362 (PMC8969518; doi:10.3389/fmicb.2022.799362)
Supplement: Supplementary file 1 [file Table_1.pdf]

Table S1. Presence or absence of Hep proteins encoded in the genome of *Richelia intracellularis* HH01. The *hep* genes have been identified in *Anabaena* sp. strain PCC 7120, and most are found in "*hep* gene islands" (Huang et al., 2005; Wang et al., 2007). Expect value from BLASTp analysis is shown in parenthesis for each detected protein.

***Anabaena hep* gene island-encoded protein**	**Homologue in *R. intracellularis* HH01**	**Comment**
Alr2825	RintHH_14280 (1 e-153)	
Alr2826	RintHH_14290 (1 e-91), RintHH_14300 (2 e-116)	Split gene in HH01
Alr2827	RintHH_14310 (0)	
Alr2828	RintHH_14320 (1 e-138)	Note gene cluster RintHH_14280 to RintHH_14310
Alr2829	Not detected	*Anabaena alr2829* mutant not demonstrated to be Fox-
Alr2830	RintHH_700 (5 e-111)	
Alr2831	RintHH_690 (0)	
Alr2832	RintHH_680 (0)	
Alr2833	RintHH_670 (0)	
Alr2834 (HepC)	RintHH_660 (5 e-28)	Lacks 52 N-terminal aas in HH01
Alr2835 (HepA)	RintHH_650 (0)	
Alr2836	RintHH_640 (2 e-14)	Lacks 150 C-terminal aas in HH01. *Anabaena alr2836* mutant not demonstrated to be Fox-
Alr2837	RintHH_640 (1 e-108)	
Alr2838	Not detected	Glycosyltransferase, Fox gene
Alr2839	RintHH_630 (9 e-19), RintHH_620 (2 e-119)	Split gene in HH01
Alr2840	RintHH_610 (1 e-145)	
Alr2841	RintHH_600 (1 e-166)	Note gene cluster RintHH_600 to RintHH_700
**Other *Anabaena* Hep proteins**		
Alr3698 (HepB)	RintHH_13820 (2 e-21), RintHH_13830 (2 e-149)	Split gene in HH01
Alr3699	RintHH_13840 (6 e-173)	Note gene cluster RintHH_13820 to RintHH_13840
All4160	RintHH_13720 (8 e-179)	

Huang G, Fan Q, Lechno-Yossef S, Wojciuch E, Wolk CP, Kaneko T, Tabata S. 2005. Clustered genes required for the synthesis of heterocyst envelope polysaccharide in *Anabaena* sp. strain PCC 7120. J Bacteriol 187(3):1114-23. doi: 10.1128/JB.187.3.1114-1123.2005.

Wang Y, Lechno-Yossef S, Gong Y, Fan Q, Wolk CP, Xu X. 2007. Predicted glycosyl transferase genes located outside the HEP island are required for formation of heterocyst envelope polysaccharide in *Anabaena* sp. strain PCC 7120. J Bacteriol 189(14):5372-8. doi: 10.1128/JB.00343-07.
