# Supplementary material for: Adaptation to an Intracellular Lifestyle by a Nitrogen-Fixing, Heterocyst-Forming Cyanobacterial Endosymbiont of a Diatom
Source: Front Microbiol. 2022 Mar 17;13:799362. doi: 10.3389/fmicb.2022.799362 (PMC8969518; doi:10.3389/fmicb.2022.799362)
Supplement: Supplementary file 2 [file Table_2.pdf]

Table S2. Presence or absence of Hgl-related proteins encoded in the genome of *R. intracellularis* HH01. The *hgl* genes have been identified in *Anabaena* sp. strain PCC 7120, and most are found in "*hgl* gene islands" (Awai and Wolk, 2007; Fan et al., 2005). Expect value from BLASTp analysis is shown in parenthesis for each detected protein.

Anabaena hgl **gene island-encoded protein**	**Homologue in *R. intracellularis* HH01**	**Comment**
All5359	RintHH_5420 (3 e-105)	
Alr5358 (HetN)	Not detected	This is a regulatory protein not present in every heterocyst former
Alr5357 (HetM)	RintHH_5430 (0)	
Alr5356 (HglA)	RintHH_5440 (0)	
Alr5355 (HglC)	RintHH_5450 (0)	
Alr5354 (HglD)	RintHH_5450 (1 e-134), RintHH_5460 (3 e-50)	Split gene in HH01 (but redundant with HglC)
Alr5353	RintHH_5470 (0)	
Alr5352	RintHH_5490 (3 e-63)	
Alr5351 ($HglE_A$)	RintHH_5500 (0)	
Asr5350	RintHH_5510 (3 e-40)	
Asr5349	Not detected	Very small protein
Alr5348 (ParB)	RintHH_5520 (0)	
All5347	RintHH_5530 (5 e-165)	
All5346	RintHH_5540 (0)	
All5345	RintHH_5550 (4 e-163)	Note gene cluster RintHH_5420 to RintHH_5550
All5344	RintHH_21930 (1 e-29)	Significance is low probably because this is a small protein
All5343	Not detected	*all5343* is an essential Fox gene in *Anabaena*
All5342	Not detected	
All5341 (HglT)	RintHH_20790 (1 e-27)	Because of low similarity, this glycosyltransferase may be unrelated to HglT
**Other *Anabaena* Hgl-related proteins**		
Alr3710 (DevB)	RintHH_11640 (2 e-170)	
Alr3711 (DevC)	RintHH_11630 (0)	
Alr3712 (DevA)	RintHH_11620 (2 e-131)	Gene cluster RintHH_11620 to RintHH_11640 encodes an Hgl exporter
Alr2887 (HgdD)	RintHH_21590 (0)	TolC-like protein

Awai K, Wolk CP. 2007. Identification of the glycosyl transferase required for synthesis of the principal glycolipid characteristic of heterocysts of *Anabaena* sp. strain PCC 7120. FEMS Microbiol Lett 266(1):98-102. doi: 10.1111/j.1574-6968.2006.00512.x.

Fan Q, Huang G, Lechno-Yossef S, Wolk CP, Kaneko T, Tabata S. 2005. Clustered genes required for synthesis and deposition of envelope glycolipids in *Anabaena* sp. strain PCC 7120. Mol Microbiol 58(1):227-43. doi: 10.1111/j.1365-2958.2005.04818.x.
