# Supplementary material for: Adaptation to an Intracellular Lifestyle by a Nitrogen-Fixing, Heterocyst-Forming Cyanobacterial Endosymbiont of a Diatom
Source: Front Microbiol. 2022 Mar 17;13:799362. doi: 10.3389/fmicb.2022.799362 (PMC8969518; doi:10.3389/fmicb.2022.799362)
Supplement: Supplementary file 3 [file Image_1.PDF]

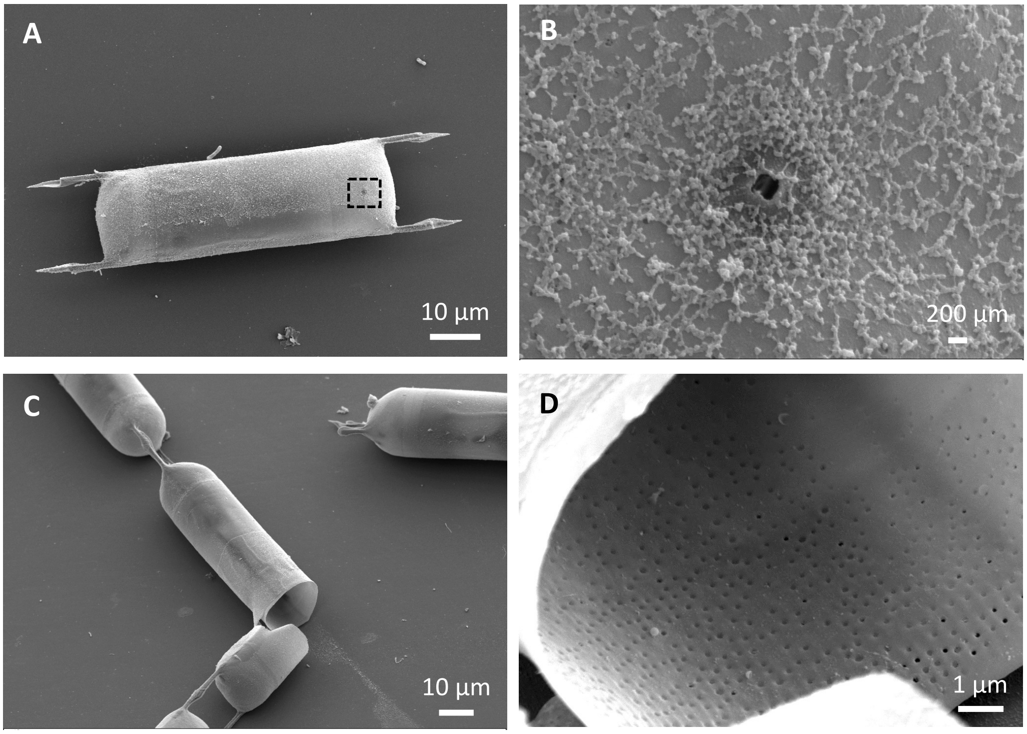


Fig. S1. Scanning electron micrographs of *H. hauckii*. (A) Visualization of a single cell. (B) Amplified square from panel A. The surface of the frustule contains a large pore that can be the external opening of the labiate process. (C) A broken cell showing the interior of the frustule. (D) Numerous small pores are visualized in the interior of the frustule.
