# Supplementary material for: Adaptation to an Intracellular Lifestyle by a Nitrogen-Fixing, Heterocyst-Forming Cyanobacterial Endosymbiont of a Diatom
Source: Front Microbiol. 2022 Mar 17;13:799362. doi: 10.3389/fmicb.2022.799362 (PMC8969518; doi:10.3389/fmicb.2022.799362)
Supplement: Supplementary file 4 [file Image_2.PDF]

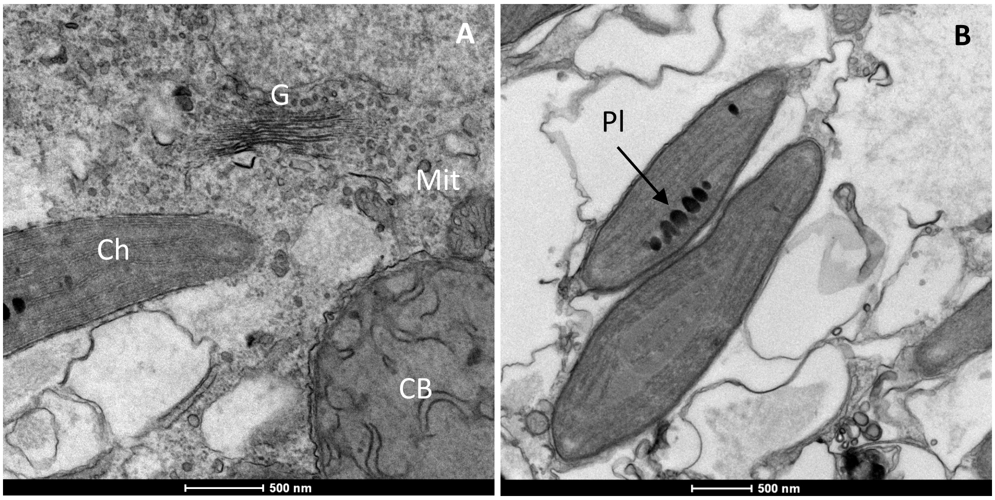

Fig. S2. Transmission electron micrographs of *H. hauckii*. (A) Visualization of some structures of a diatom cell including an endosymbiotic cyanobacterium. G, Golgi bodies; Ch, chloroplast; Mit, mitochondrion; CB, cyanobacterial cell. (B) Magnified view of a zone of the diatom shown in Fig. 2C including two chloroplasts, one of them containing plastoglobules (Pl).
