# Supplementary material for: Adaptation to an Intracellular Lifestyle by a Nitrogen-Fixing, Heterocyst-Forming Cyanobacterial Endosymbiont of a Diatom
Source: Front Microbiol. 2022 Mar 17;13:799362. doi: 10.3389/fmicb.2022.799362 (PMC8969518; doi:10.3389/fmicb.2022.799362)
Supplement: Supplementary file 5 [file Image_3.PDF]

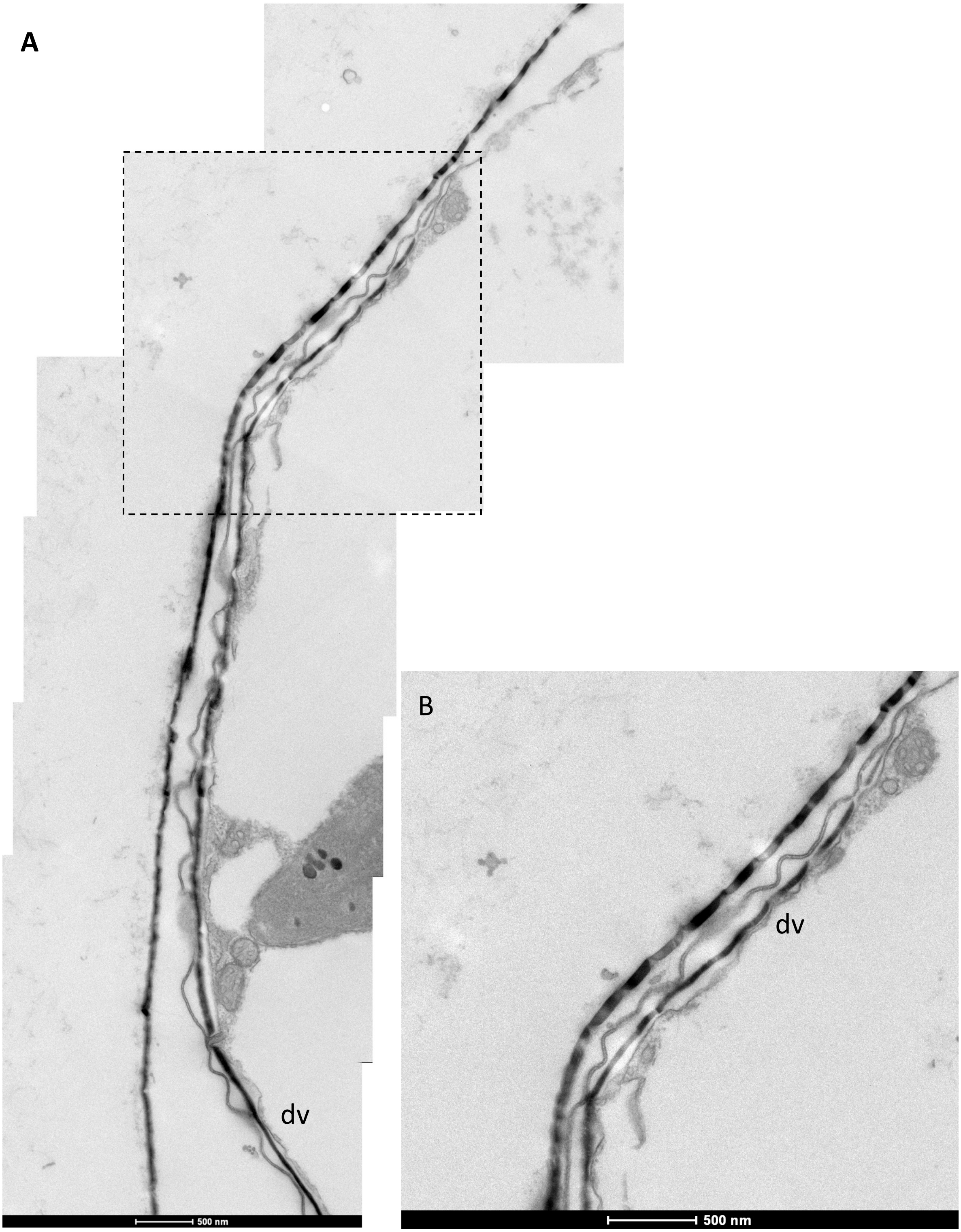

Fig. S3. Transmission electron micrographs of *H. hauckii.* (A) Magnified view of the diatom cell shown in Fig. 2D. Note that the developing valve (dv) is within membranes. (B) Magnified view of the square indicated in panel A.
