# Supplementary material for: Adaptation to an Intracellular Lifestyle by a Nitrogen-Fixing, Heterocyst-Forming Cyanobacterial Endosymbiont of a Diatom
Source: Front Microbiol. 2022 Mar 17;13:799362. doi: 10.3389/fmicb.2022.799362 (PMC8969518; doi:10.3389/fmicb.2022.799362)
Supplement: Supplementary file 6 [file Image_4.PDF]

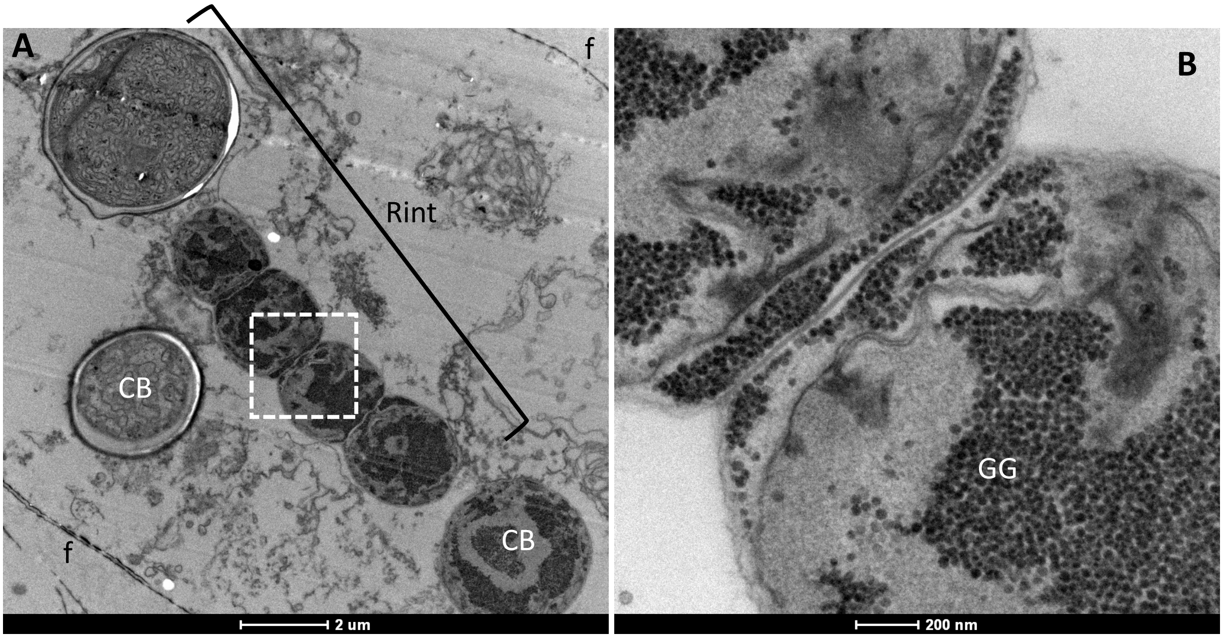

Fig. S4. Transmission electron micrograph of a *H. hauckii* cell containing *R. intracellularis*. (A) Longitudinal section of a cyanobacterial filament (Rint) and two other possible cyanobacterial cells (CB) are observed within a diatom cell; f, frustule. (B) Magnified view of a section (dotted square) of the cyanobacterial filament showing numerous glycogen granules (GG) in the cells.
