# Supplementary material for: Adaptation to an Intracellular Lifestyle by a Nitrogen-Fixing, Heterocyst-Forming Cyanobacterial Endosymbiont of a Diatom
Source: Front Microbiol. 2022 Mar 17;13:799362. doi: 10.3389/fmicb.2022.799362 (PMC8969518; doi:10.3389/fmicb.2022.799362)
Supplement: Supplementary file 7 [file Image_5.PDF]

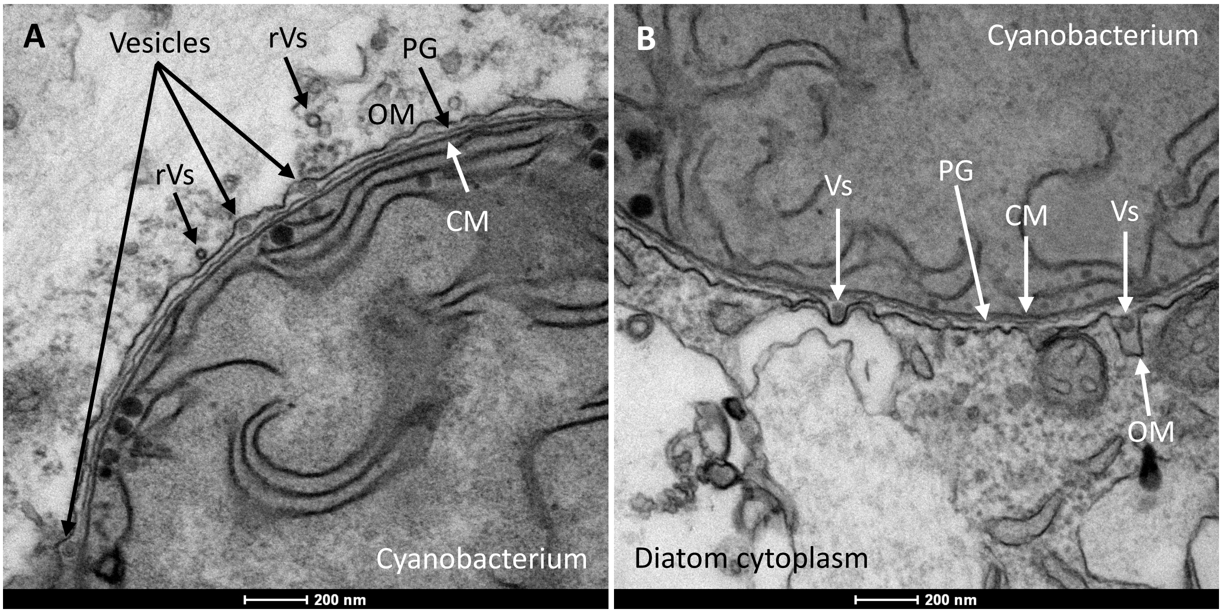

Fig. S5. Transmission electron micrograph of the *R. intracellularis* envelope and vesicles. (A) Magnified view of a section of the cyanobacterium shown in Fig. 4B detailing envelope structures: cytoplasmic membrane (CM), peptidoglycan layer (PG) and outer membrane (OM). Note the presence of some vesicles in the periplasmic space. rVs, possible recently released vesicles. (B) Cell envelope components of a cyanobacterial cell showing two vesicles within outer membrane evaginations.
