# Supplementary material for: Adaptation to an Intracellular Lifestyle by a Nitrogen-Fixing, Heterocyst-Forming Cyanobacterial Endosymbiont of a Diatom
Source: Front Microbiol. 2022 Mar 17;13:799362. doi: 10.3389/fmicb.2022.799362 (PMC8969518; doi:10.3389/fmicb.2022.799362)
Supplement: Supplementary file 8 [file Image_6.PDF]

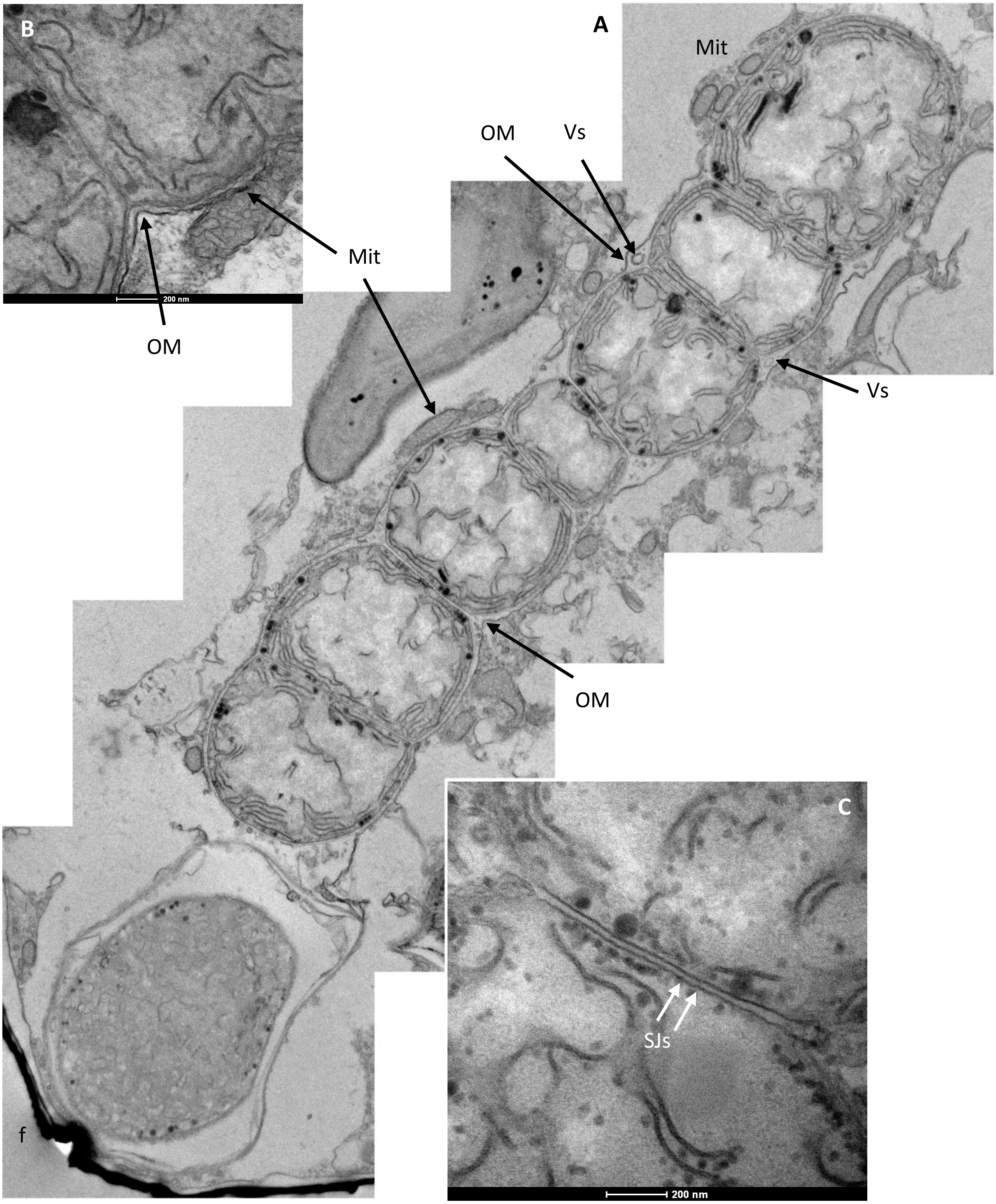

Fig. S6. Transmission electron micrographs of a filament of *R. intracellularis* endosymbiotic in *H. hauckii*. (A) Montage showing the continuity of the outer membrane (OM) along the filament. Mitochondria (Mit) nearby the cyanobacterium and periplasmic vesicles (Vs) are also observed; f, frustule. (B) Micrograph of part of a *R. intracellularis* filament showing very clearly the continuous OM and a mitochondrion closely associated to the OM of the cyanobacterium. (C) Septum between two vegetative cells of *R. intracellularis* with evidence of the presence of septal junctions (SJs).
