# Supplementary material for: Adaptation to an Intracellular Lifestyle by a Nitrogen-Fixing, Heterocyst-Forming Cyanobacterial Endosymbiont of a Diatom
Source: Front Microbiol. 2022 Mar 17;13:799362. doi: 10.3389/fmicb.2022.799362 (PMC8969518; doi:10.3389/fmicb.2022.799362)
Supplement: Supplementary file 9 [file Image_7.PDF]

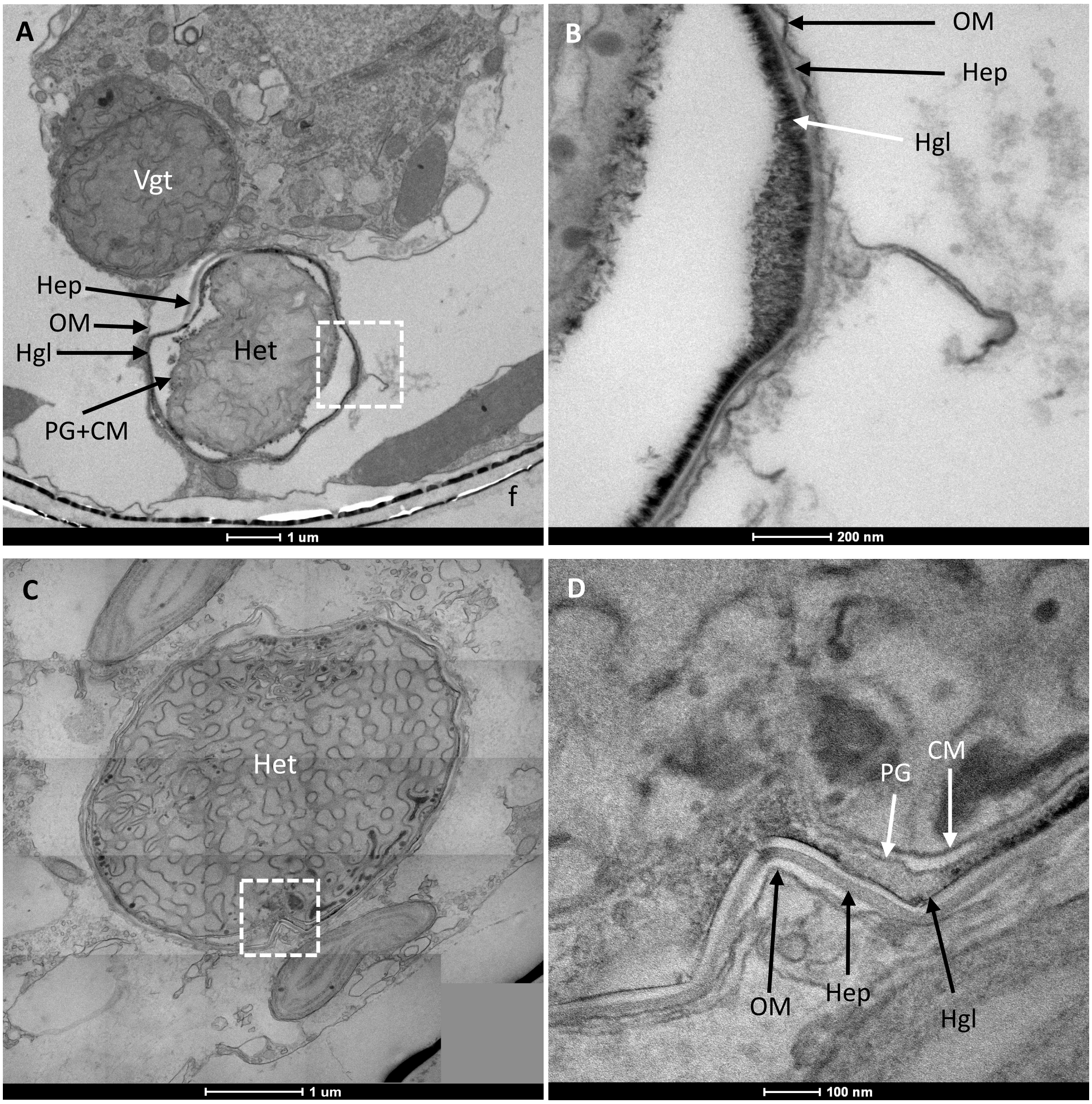

Fig. S7. Transmission electron micrographs of *R. intracellularis* endosymbiotic in *H. hauckii*. (A) Part of a diatom cell is shown in which one heterocyst (Het), its adjacent vegetative cell (Vgt) and a fragment of the next cell in the filament are discernible; f, diatom frustule. The heterocyst outer membrane (OM) is the outer-most envelope layer and is continuous with that of the adjacent vegetative cell. Internal to the OM there are a layer putatively identified as heterocyst polysaccharide layer (Hep) and the very well labeled layer putatively identified as heterocyst glycolipid layer (Hgl). The shrinked heterocyst cytoplasm should be surrounded by the cytoplasmic membrane (CM) and a peptidoglycan layer (PG). (B) Detail (dotted square in panel A) of the heterocyst envelope showing the outer membrane (OM) and possible Hep and Hgl layers. (C) Transverse section of a heterocyst. (D) Detail (dotted square in panel C) of the heterocyst envelope showing the cytoplasmic (CM) and outer (OM) membranes and tentatively identified PG, Hep and Hgl layers.
