# Supplementary material for: Adaptation to an Intracellular Lifestyle by a Nitrogen-Fixing, Heterocyst-Forming Cyanobacterial Endosymbiont of a Diatom
Source: Front Microbiol. 2022 Mar 17;13:799362. doi: 10.3389/fmicb.2022.799362 (PMC8969518; doi:10.3389/fmicb.2022.799362)
Supplement: Supplementary file 10 [file Image_8.PDF]

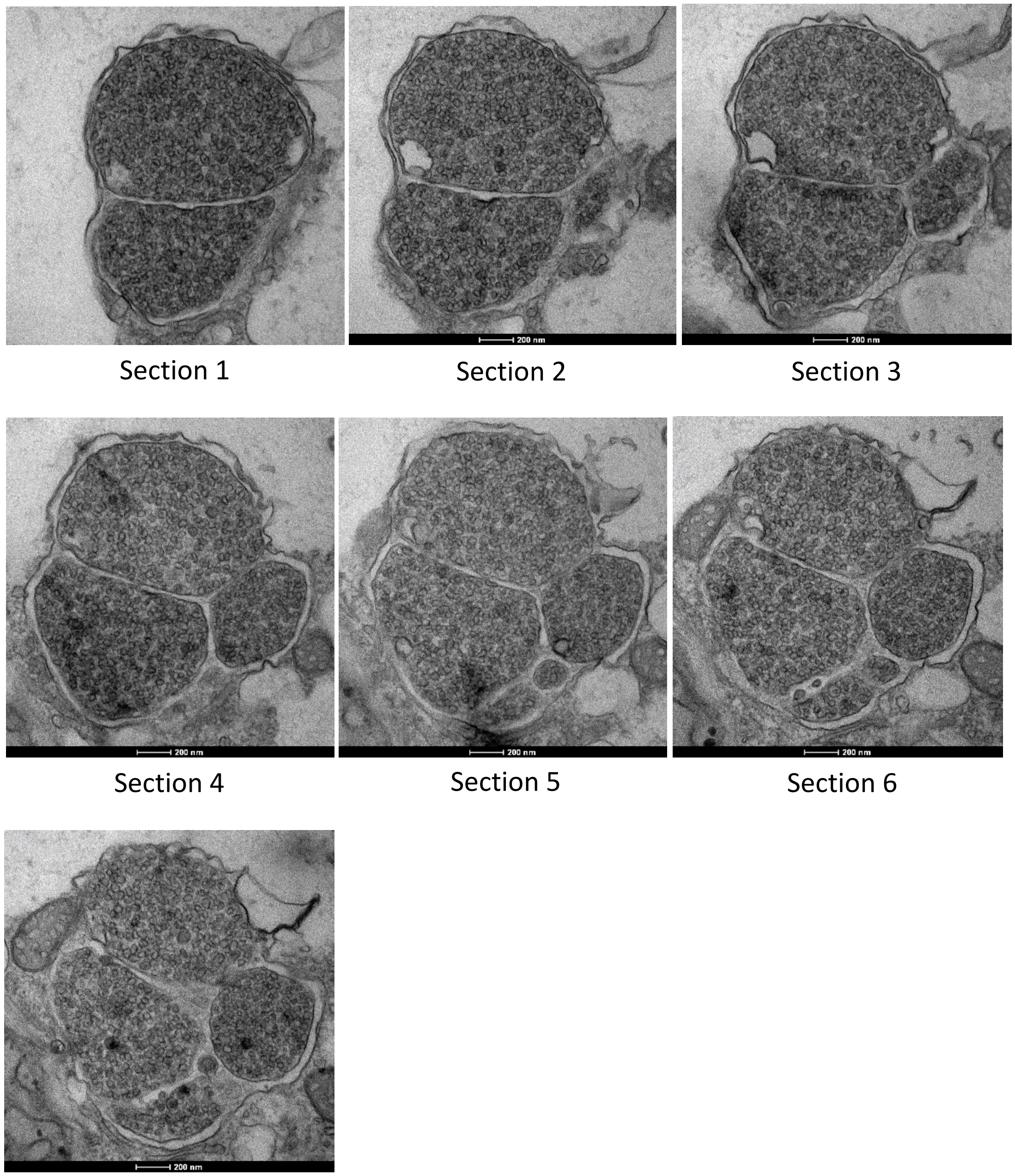

Fig. S8. Transmission electron micrographs of a putative second endosymbiont in *Hemiaulus hauckii.* The whole series of 70-nm sections in which the second endosymbiont was observed is presented.
