# Supplementary material for: Adaptation to an Intracellular Lifestyle by a Nitrogen-Fixing, Heterocyst-Forming Cyanobacterial Endosymbiont of a Diatom
Source: Front Microbiol. 2022 Mar 17;13:799362. doi: 10.3389/fmicb.2022.799362 (PMC8969518; doi:10.3389/fmicb.2022.799362)
Supplement: Supplementary file 11 [file Image_9.PDF]

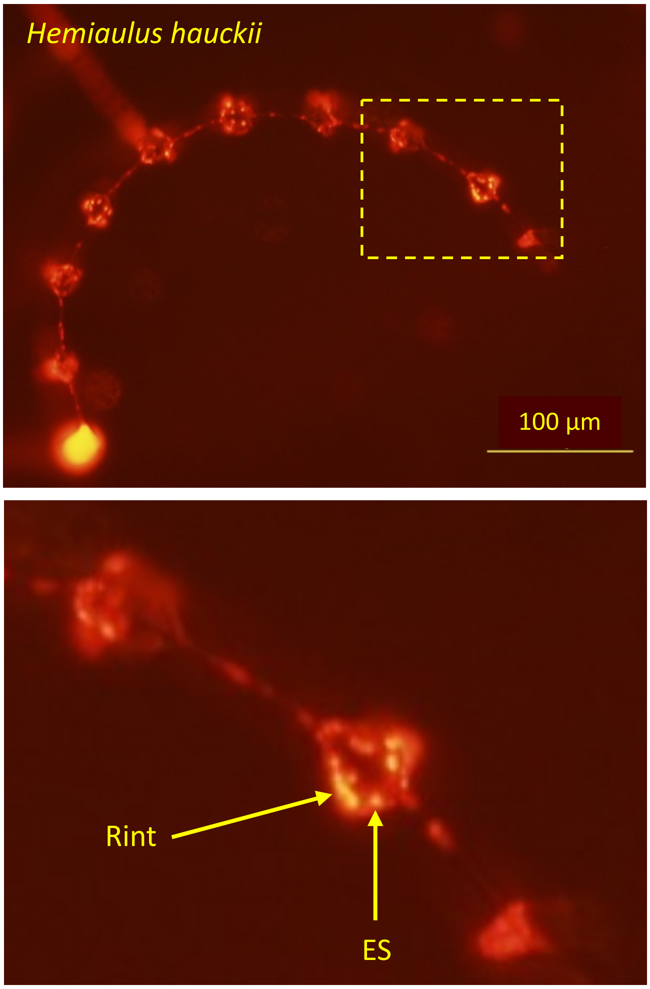

Fig. S9. Epifluorescence micrographs of *H. hauckii* showing several possible endosymbionts. Samples collected from the Amazon River plume were visualized by epifluoresce microscopy (Pyle et al., 2020). Excitation/emission wavelengths for the epifluorescent filters used were 450 nm/680 nm (chlorophyll *a* [red fluorescence]) and 490 nm/565 nm (phycoerythrin [orange/yellow fluorescence]). The upper micrograph shows a chain of *H. hauckii* cells. The lower micrograph shows, magnified, the square area indicated in the upper micrograph. Note the presence in a diatom cell of several fluorescent spots, some of which can correspond to a filament of *R. intracellularis* (Rint) and other to a non-filamentous cyanobacterium (ES for endosymbiont).
